# BRIDGE – A Visual Analytics Web Tool for Barley Genebank Genomics

**DOI:** 10.3389/fpls.2020.00701

**Published:** 2020-06-11

**Authors:** Patrick König, Sebastian Beier, Martin Basterrechea, Danuta Schüler, Daniel Arend, Martin Mascher, Nils Stein, Uwe Scholz, Matthias Lange

**Affiliations:** ^1^Leibniz Institute of Plant Genetics and Crop Plant Research (IPK), Seeland, Germany; ^2^German Centre for Integrative Biodiversity Research (iDiv), Leipzig, Germany; ^3^Center for Integrated Breeding Research, Georg-August University, Göttingen, Germany

**Keywords:** barley, plant genetic resources, genebank genomics, visual analytics, data visualization, phenotyping, genotyping, data warehouse

## Abstract

Genebanks harbor a large treasure trove of untapped plant genetic diversity. A growing world population and a changing climate require an increase in the production and development of stress resistant plant cultivars while decreasing the acreage. These requirements for improved plant cultivars can be supported by the broader exploitation of plant genetic resources (PGR) as inputs for genomics-assisted breeding. To support this process we have developed BRIDGE, a data warehouse and exploratory data analysis tool for genebank genomics of barley (*Hordeum vulgare* L.). Using efficient technologies for data storage, data transfer and web development, we facilitate access to digital genebank resources of barley by prioritizing the interactive and visual analysis of integrated genotypic and phenotypic data. The underlying data resulted from a barley genebank genomics study cataloging sequence and morphological data of 22,626 barley accessions, mainly from the German Federal *ex situ* genebank. BRIDGE consists of interactively coupled modules to visualize integrated, curated and quality checked data, such as variation data, results of dimensionality reduction and genome wide association studies (GWAS), phenotyping results, passport data as well as the geographic distribution of germplasm samples. The core component is a manager for custom collections of germplasm. A search module to find and select germplasm by passport and phenotypic attributes is included as well as modules to export genotypic data in gzip-compressed variant call format (VCF) files and phenotypic data in MIAPPE-compliant ISA-Tab files. BRIDGE is accessible at the following URL: https://bridge.ipk-gatersleben.de.

## Introduction

Cereal grasses like barley, rye and wheat are the main nutrition source for human calorie intake in the world (FAO, 2018). With its diploid genome, inbreeding feature and in comparison to its relatives wheat and rye rather small genome size of 5.1 Gbp, barley is an excellent model for basic and applied research in the *Triticeae* tribe. Large collections of plant genetic resources for food and agriculture (PGRFA) of diverse barley genotypes and phenotypes have been described in the literature (Ullrich, 2010). The German Federal *ex situ* genebank hosted at the Leibniz Institute of Plant Genetics and Crop Plant Research in Gatersleben hosts more than 22,000 barley accessions consisting of wild relatives, landraces, and breeding material collected over the past 70 years (Nagel et al., 2009; González et al., 2018; Milner et al., 2019). In prospect of the upcoming challenges of continued growth of the world population, climate change, and increasing scarcity of resources like arable land, water, and fertilizers enhances the pressure on agriculture to provide humankind with sufficient food (Pachauri et al., 2015; FAO, 2018). PGRFA hold a promise for the way of responding to this pressure through crop improvement and yield increase per hectare. The necessary continued crop improvement can be achieved by modern plant breeding methods like marker assisted selection (Varshney et al., 2005; Crossa et al., 2017), reverse breeding (Dirks et al., 2009), and genome engineering/editing (Voytas and Gao, 2014). These methods allow to increasingly benefit from the putative advantageous alleles of the PGRFA, which have not yet been used in recent breeding efforts. To accompany this process, information systems are needed that integrate the extensive amounts of derived multi-omics data and make them accessible and available in a FAIR manner (Wilkinson et al., 2016). Based on the integrated datasets of these information systems, decisions for the optimization of the breeding process and curation of passport data can be derived.

Due to constantly decreasing costs for DNA sequencing, ever more extensive sequencing projects can be carried out at constant or even further decreasing costs (Shendure and Ji, 2008; Shendure et al., 2017). This leads to constantly increasing amounts of raw, processed, and analyzed data that has to be stored, curated and integrated (Muir et al., 2016). Advancement in the field of sequencing have led to different experimental setups like resequencing on a population scale with various complexity reduction methods to derive molecular fingerprints of whole breeding panels (Varshney et al., 2009; Poland and Rife, 2012; Jarquín et al., 2014). Furthermore, even more ambitious projects have been initiated to generate many different reference genome assemblies to fully describe the pangenome (Weigel and Mott, 2009; Hirsch et al., 2014; Vernikos et al., 2015).

The field of non-invasive plant imaging has also seen tremendous advancements, with fully automated imaging systems able to produce multi- and hyperspectral images (Fiorani and Schurr, 2013; Coppens et al., 2017; Pieruschka and Schurr, 2019). However, the produced data for phenotyping experiments can in general be more heterogeneous than for genotyping. Expressed phenotypes can vary not only between genotypes but also between cell types (Houle et al., 2010). In addition, because of the high plasticity of plants, where different environments can trigger a single genotype to express different phenotypes, it is imperative to collect a multitude of heterogeneous data (Bolger et al., 2019).

There are well established and community accepted repositories, data deposition, and data exchange formats for genomic data, such as the European Nucleotide Archive (ENA) (Silvester et al., 2018), the National Center for Biotechnology Information (NCBI) (NCBI Resource Coordinators, 2017), and the DNA Data Bank of Japan (DDBJ) (Tateno, 2002), which are part of the International Nucleotide Sequence Database Collaboration (Cochrane et al., 2016). In contrast, there is yet no comparable central, dedicated database for phenotypic data, especially for plants. Rather, there are a large number of repositories with different specific focuses and data formats (Coppens et al., 2017). To supersede many different and incompatible text based formats for data storage and data exchange an international committee has agreed upon recommendations for a set of minimal information about plant phenotyping experiments (MIAPPE) (Krajewski et al., 2015; Ćwiek-Kupczyńska et al., 2016). An established way to digitize the results of biological and life science experiments for storage is the ISA framework (Sansone et al., 2012) and in particular the “ISA-Tab” file format (Rocca-Serra et al., 2010). In the plant science community, there is a common effort to establish the usage of MIAPPE-compliant ISA-Tab files for the export and transfer of data for plant phenotyping experiments (Krajewski et al., 2015).

The combination of genomic and phenotypic characteristics form a high-dimensional data space. Traditional genebank web portals and germplasm data warehouses, like e.g., GBIS (Oppermann et al., 2015) and EURISCO (Weise et al., 2017), have so far not focused on the visualization and explorative data analysis of integrated, high-dimensional genotypic and phenotypic datasets of entire germplasm collections. This is also the case because the specific functional scope of such genebank portals has historically been developed primarily with regard to basic genebank management tasks like seed management and reproduction of PGRs, but is not geared toward the integration or interoperability to multi-omics data repositories, visual analytics or exploratory data analysis.

Here, we present BRIDGE, a web application built to help explore and analyze the results of research data from a comprehensive barley geno- and phenotyping project of a panel of 22,626 barley (*Hordeum vulgare* L.) accessions. Genotypic data were determined for 22,621 accessions and stored in a relational database management system (RDBMS). Accessing this data source using the web frontend, the user is able to navigate between phenotypic traits, genetic and genomic information from genotyping-by-sequencing derived single nucleotide polymorphism (SNP) profiles, and can correlate them with passport information in several easy-to-understand graphical visualizations of the data. Furthermore, BRIDGE is directly connected to the information management system of the German Federal *ex situ* genebank hosted at the Leibniz Institute of Plant Genetics and Crop Plant Research (IPK) in Gatersleben, Germany. Users are able to order seed material based on their custom germplasm selections.

## Materials and Methods

### Application Architecture

**FIGURE 1:**
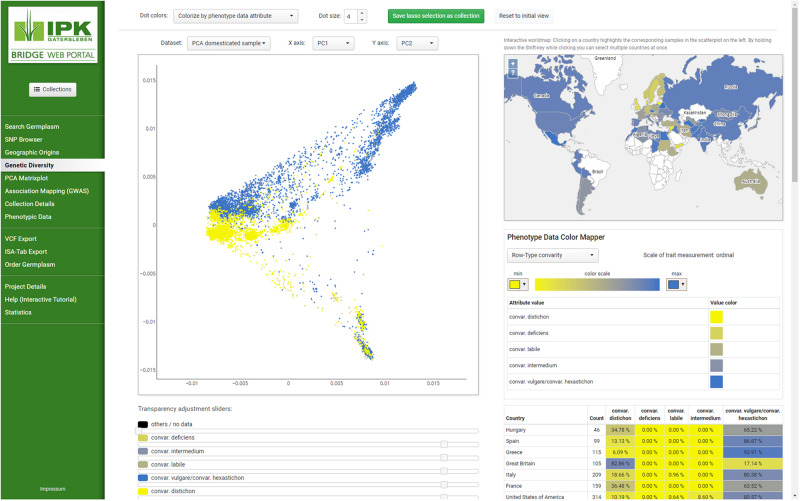
Screenshot of the layout and BRIDGE user interface. Navigating to different parts of the page can be done by selecting the menu items on the left which are clustered into thematic groups and separated by horizontal lines to give users a visual structure. Changing from one menu item to the next does not change the appearance of the web portal, whereby both the layout remains intact and the applied settings are transferred to the entire page. This behavior is compliant to the single-page application (SPA) paradigm. As example “Genetic Diversity” from the navigation menu has been selected to present the colorization of subsets in both PCA plot and the distribution of accessions on a linked world map. Parameters of the visualization can be changed with the buttons, sliders and select boxes throughout the page. Detailed information regarding the selected subsets can be found underneath the plots.

**FIGURE 2:**
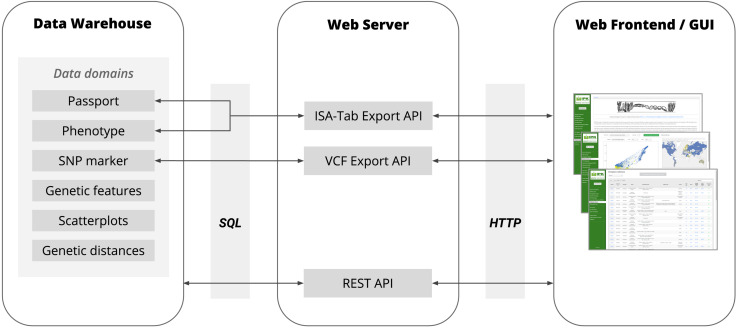
The basic architecture of BRIDGE showing the flow of data between the Data Warehouse, Web Server and Web Frontend with all available data domains. The Data Warehouse consists of several SQL tables in the in-house ORACLE RDBMS. The communication between the Data Warehouse and Web Server is based on the JDBC interface using SQL as query language. The Web Server provided a REST API for the Web Frontend to deliver the data points for all data visualization modules and to respond to requests for search queries initiated by a user via the Web Frontend. Furthermore, the Web Server provides APIs for the export of data of genetic variants as VCF files and the export of phenotypic data in MIAPPE-compliant ISA-Tab archives. The communication between the Web Server and Web Frontend is based on the HTTP protocol.

BRIDGE follows the client-server architecture model. The client is built as a web frontend (Figure 1) based on the concept of single-page applications (SPA) utilizing HTML, CSS, JavaScript, and established web technology standards like a RESTful API for communication with the server-side part of the application. The web frontend is implemented in a model-view-controller (MVC) pattern (Leff and Rayfield, 2001) based modular architecture. The graphical user interface uses jQuery in version 3.2.1 (De Volder, 2006) and Vue.js in version 2.6.10 (Filipova, 2016) for the rendering of user interface widgets, the interaction with the Document Object Model (DOM) and the handling of events like user inputs. The interactive scatterplots are implemented with the Plotly.js library in version 1.43.2 (Sievert et al., 2017), which uses WebGL for hardware-accelerated high-performance rendering of plots with millions of data points. The world map was implemented with OpenLayers in version 5.3.0 (Hazzard, 2011). The client consists of a core library, which is extended by additional loosely coupled modules for each visualization or data export feature. The core library provides baseline functionality like the management of sample collections across the different modules. Due to the usage of advanced web technology standards such as HTML5 and CSS3 in the frontend, access to the webportal requires recent versions of web browsers such as Mozilla Firefox, Google Chrome, Apple Safari or Microsoft Edge. The server-side backend of BRIDGE was built as a Java Virtual Machine application using the Grails web application framework in version 3.3.8 (Smith and Ledbrook, 2009). The architecture of the BRIDGE system is shown in Figure 2.

### Basic Usability Concepts

**FIGURE 3:**
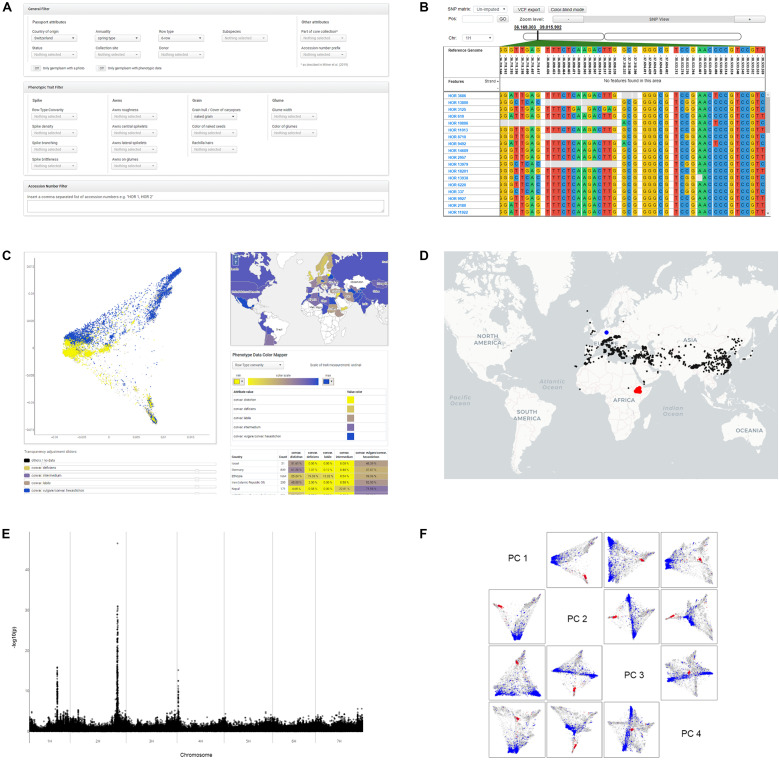
Screenshots as illustrations of the most important search and visualization features, which can be used as an entry point for exploratory data analysis. **(A)** Data Filters: In the “Search Germplasm” panel, the user can search for germplasm by filtering passport attributes, phenotypic traits and SNP markers. **(B)** Genomic Diversity Visualization: In the integrated SNP browser users can inspect and subset the SNP matrices visually for different collections of germplasm. **(C)** Combined Interactive Visualizations: This tool enables the correlation of results of dimensionality reduction algorithms like PCA or t-SNE on the SNP data with countries of origin and phenotypic traits of the germplasm. **(D)** Interactive World Map: This tool allows the user to create lasso selections of geo-localized germplasm or to highlight user-defined germplasm collections with their specific tagging colors. **(E)** Manhattan Plots: This tool provides interactive plots of GWAS analysis results where each SNP data point is linked to the SNP browser. The user can click on a SNP data point and is then automatically guided to the corresponding genomic location in the SNP browser. **(F)** PCA Scatterplot Matrix: This visualization tool allows visual inspection of the first four principal components while highlighting user-defined germplasm collections with their specific tagging colors. It also allows to save custom lasso-selection of data points as a “named collection” of germplasm.

**FIGURE 4:**
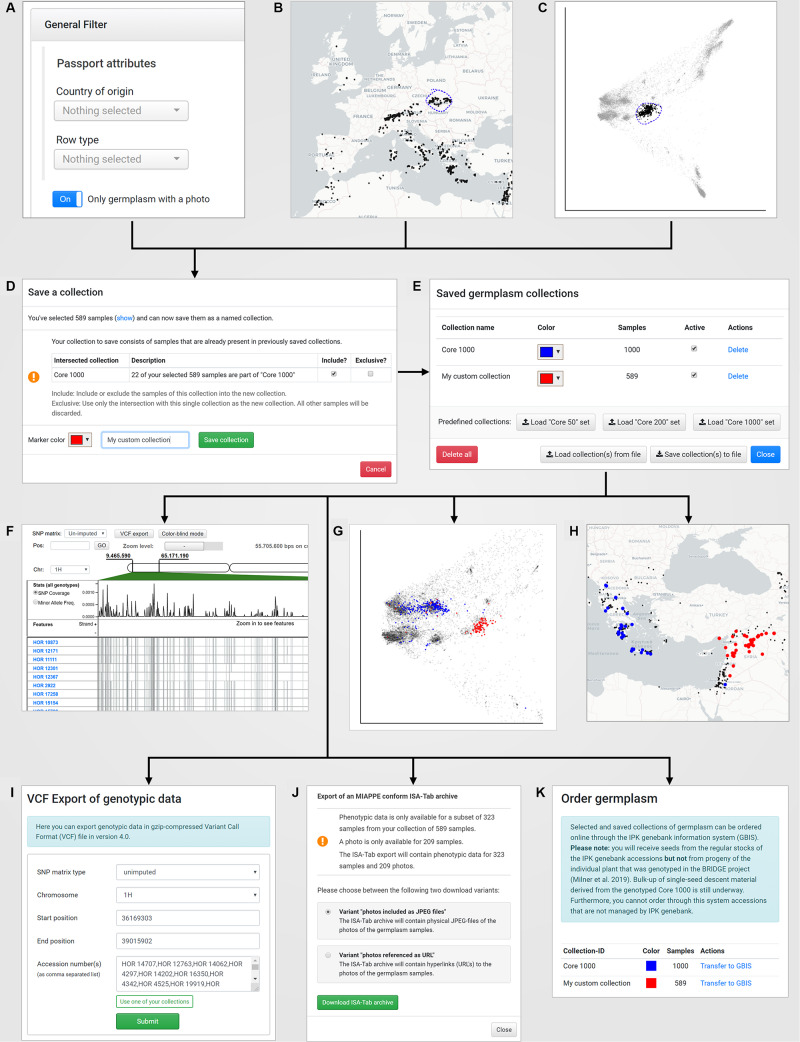
The concept of “named collections” in combination with the visual analytics concept of “interactive brushing and linking:” A new germplasm collection can be created by **(A)** defining filters on passport records (under “Search Germplasm”), **(B)** a lasso selection in the world map (under “Geographic Origins”), **(C)** a lasso selection in a PCA plot (under “Genetic Diversity”). The save dialog **(D)** allows to store the current collection with a custom name und tagging color. Furthermore, the save dialog automatically detects intersections of the current selected germplasm samples with already existing germplasm collections and provides a function to add, subtract or intersect the sample lists. After saving, the new collection is available in the “Saved germplasm collections” dialog that is located in the menu “Collections” **(E)** and can be reused for application-wide visualization in the SNP browser **(F)**, the PCA plots for exploration of the genetic diversity space **(G)** and the world map **(H)**. Also, the SNP data for that collection can be exported to a VCF file **(I)**, the phenotypic and passport data including pictures of the selected accessions in the collection can be downloaded as a MIAPPE compliant ISA-Tab archive **(J)** and finally the germplasm for the collection can be ordered online from the IPK genebank information system **(K)**.

BRIDGE uses a concept of “named collections” of germplasm samples as the foundation for an interactive and exploratory data analysis workflow. A named collection is a user-defined, private and re-usable set of germplasm samples that can feed into the modules for visual analytics and data export. Each collection can be assigned an individual color, which is later used in the data visualization as a tagging color. Collections can be compiled by saving the result of a germplasm search (Figure 3A) or by saving the result of a lasso selection in a scatterplot (Figure 3C) or on the world map (Figure 3D). The user is notified if a new collection intersects with a previously saved collection (Figure 4D) and can decide whether to include or exclude the shared samples. It is also possible to save only the intersection itself while omitting all other samples, allowing the iterative refinement of collections.

Another concept used in BRIDGE is the synchronization of visualization modules called “interactive brushing and linking,” a technique that combines different visualization methods to achieve a greater benefit compared to the standalone usage of single visualization methods (Keim, 2002). Basically, a change of parameters in one visualization directly influences the visualization of data in other visualizations. In this context, brushing is understood as highlighting the same subset of data in dynamically coupled visualizations. This functionality is implemented by an automatic color tagging of data points of the user-defined germplasm sample collections in the various visualization modules (Figure 4).

### Available Data

The provided data in BRIDGE are the results of the study published by Milner et al. (2019) who compiled molecular passport data for the entire barley germplasm collection of the IPK genebank and several hundred additional accessions from genebanks that were incorporated in the corresponding research project. The available data in BRIDGE consists of passport data of 22,626 germplasm samples, phenotypic data of 9,527 germplasm samples, SNP matrices, visualization of the genetic diversity space by PCA and t-SNE (Maaten and van der Hinton, 2008) and Manhattan plots of GWAS results. As a result of ongoing research and maintenance, passport data are continuously curated by the genebanks and can therefore vary over time. To ensure consistency with the source data of the study of Milner et al. (2019), the passport data used in BRIDGE is a snapshot taken at the beginning of the study. A categorized summary of the data stock is listed in Table 1. The available attributes and completeness of passport records is listed in Table 2. Additional attributes are the affiliation of a germplasm sample to one of the three Core sets, the accession number prefix and the row type derived from the full botanical name of the accession. This row type attribute is present in almost all germplasm samples, but may contain errors due to data problems in the full botanical names of historical passport data. It is important to note that this row type, which is derived from passport data, does not have to be the same as the phenotyped row type (for example because of intra-accession heterogeneity or a mislabeling of the subspecies attribute). A detailed tabular summary of all available phenotypic traits with all possible values and the count of germplasm samples for each distinct trait value is listed in Table 3. We note that the number of observed phenotypes and passport information is very unbalanced. This is due to the limited project resources in terms of available working time for manual phenotyping.

**TABLE 1:** Numbers of germplasm samples with specific available data attributes.

Available data	Number of data records
Germplasm samples with passport data	22,626
Genotyped germplasm samples (available in VCF file)	22,621
Germplasm samples with at least one observed phenotype	9,527
Germplasm samples with spike photographs	6,162
Germplasm samples with geographical (GPS) coordinates	2,862

**TABLE 2:** Available passport data attributes with corresponding MCPD codes (Multi-Crop Passport Descriptor Codes) (Alercia et al., 2015) and data completeness.

Passport attribute	MCPD code	Data completeness
Country of origin	ORIGCTY	88.64%
Subtaxon/Subspecies	SUBTAXA	99.99%
Biological status of accession	SAMPSTAT	99.57%
Donor institute	DONORCODE	N/A
Location of collecting site	COLLSITE	29.27%
Full botanical name	N/A	95,15%
Annual growth habit (barley specific)	N/A	90.21%

*For the attribute “Donor institute” the specification of data completeness makes no sense and is therefore not provided.*

**TABLE 3:** Observed phenotypic traits of barley spikes and number of accessions per trait value in the study panel.

Phenotypic trait	Possible values	# Accessions
Row-type convarity	convar. *vulgare*/convar. *hexastichon*	5,572
	convar. *distichon*	3,030
	convar. *deficiens*	411
	convar. *labile*	293
	convar. *intermedium*	207
Spike density	Lax	7,804
	Middle	1,483
	Dense	234
Spike branching	Unbranched	9,367
	Branched	153
Spike brittleness	No	9,517
	Yes	4
Grain hull / Cover of caryopses	Covered grain	8,630
	Naked grain	882
Color of naked seeds	Yellow	726
	Between black and brown	57
	Black	50
	Green	32
	Purple	8
	Other	9
Length of rachilla hairs	Long	6,019
	Short	3,474
Awns roughness	Rough	8,926
	Smooth	421
Presence of awns central spikelet	Awnless or very short awns (tips)	71
	Awns short (up to spike length)	450
	Awns long (1.5–3 times spike length)	8,850
	Sessile hoods (sessile or on short)	27
	Elevated hoods (Hood over 1 cm long awns shaped stems)	19
	Hoods with end awn	19
	Elevated hoods with end awn	34
Presence of awns lateral spikelets	Awnless or very short awns (tips)	203
	Awns short (up to spike length)	419
	Awns long (1.5–3 times spike length)	5,276
	Sessile hoods (sessile or on short)	19
	Elevated hoods (Hood over 1 cm long awns shaped stems)	17
	Hoods with end awn	15
	Elevated hoods with end awn	20
Presence of awns on glumes	Awnless or very short awns (tips)	8,725
	Awns short (up to spike length)	785
	Awns long (1.5–3 times spike length)	10
	Sessile hoods (sessile or on short)	0
	Elevated hoods (Hood over 1 cm long awns shaped stems)	0
	Hoods with end awn	0
	Elevated hoods with end awn	1
Glume width	All glumes are narrow (<1 mm)	5,959
	All or some glumes are broad (1–2 mm)	56
Color of glumes	Yellow	5,614
	Gray	122
	Black	115
	Brown	92
	Purple	71
	Green	1
	Other	4

Genetic variation data are present in the form of two SNP matrices: one with unimputed data of 187,713 SNPs and 22,621 genotypes and another with imputed data of 306,049 SNPs and 20,458 genotypes. SNP positions are given relative to the first version reference sequence assembly of cv. Morex (Mascher et al., 2017). As data for the genome annotation in the SNP browser the data sets of structural and functional information of the International Barley Sequencing Consortium was used (IBSC,2016a, b).

Results of GWAS are available for the following analyzed traits:

• Grain hull (covered vs. naked),
• Row-type (two-rowed vs. six-rowed),
• Awn roughness,
• Resistance to Barley Mild Mosaic Virus (BaMMV),
• Flowering time.

The dimensionality reduction of the SNP data with the t-SNE algorithm was calculated using the function *TSNE* of the *scikit-learn* package (Pedregosa et al., 2011) for Python. Except for the parameter “perplexity” the function was called with default values for all parameters. The parameter “perplexity” was varied to shift between local and global structure preservation. To feed the *TSNE* function, the VCF file was converted into a NumPy array (van der Walt et al., 2011) using the function *read_vcf* of the *scikit-allel* package (Miles et al., 2019).

### Functionalities and Features

BRIDGE consists of data domain specific search, visualization and export modules. Their functionalities and features will be described in the following.

#### Sample Collection Manager

The sample collection manager (Figure 4E) stores user-defined germplasm collections with a title and a specific color that is later used as a tagging color in the data visualization modules (Figures 3C, 4D–F). There are predefined collections such as the “Core 50,” “Core 200,” and “Core 1000” collections as described in Milner et al. (2019) can be loaded directly via dedicated buttons. Each collection can be toggled by a checkbox to show or hide the collection in all visualization modules simultaneously. All collections are saved for private access only in the IndexedDB of the user’s browser and persist after leaving the web portal and even computer shutdowns (Kimak and Ellman, 2015), unless the private browsing options are used. This approach makes it possible to avoid server-side storage of potentially sensitive data and a user authentication mechanism that would require prior registration. The individual set of collections can also be exported to the local filesystem as a JSON-document and reversely be loaded again.

#### Passport, Phenotypic, and Variant Data Based Germplasm Search

BRIDGE provides a combined search functionality for passport, phenotypic, and genotypic data. Users are able to filter for any combination of passport, phenotypic or variant data (SNPs) or a combination of all three data domains (Figure 3A). To avoid complex database queries involving computation-intensive SQL joins in the database backend, the three data domains are queried separately, and an intersection of the sample IDs of all three result sets is then sent to the frontend. The intersection is the result set that fulfills the search conditions on all three data domains. In addition, the direct search for germplasm via the accession number is possible. The search result can be exported as a CSV file.

#### SNP Browser

The SNP browser, first described in Basterrechea (2017), allows the visual exploration of the SNP matrices over all sequenced germplasm samples (Figure 3B). The user interface consists of three parts. The first part with the navigation in the upper area displays the current genome coordinates and allows to change them. The displayed SNP matrix can be changed via a dedicated selector. Furthermore, there are buttons to zoom in and out, to trigger a VCF export and to toggle a “colorblind mode” for people with red-green visual impairment. The second part below the navigation has a variable display area whose display content depends on the current zoom level. At the maximum zoom level, the nucleotides of the reference genome are displayed. When zooming out, the display changes to show SNP density, minor allele frequency (calculated across all germplasm samples) and a track with genes in genomic windows of variable size. The third part in the lower area of the user interface shows the genotypes of the currently selected collection as tracks. At maximum zoom level each track shows the distinct variants for all marker positions in the manner of a slice of the SNP matrix. When zooming out, the track visualization changes to show SNP coverage for each genotype in the current genomic window. The user can move the viewport and thus the sample list and genomic window by dragging with the mouse pointer. The default zoom level is the nucleotide level view where one can see single SNPs in a linear arrangement. The export of SNP matrices in variant call format (VCF) (Danecek et al., 2011) can be directly triggered from the current viewport of the SNP browser. Then the current list of germplasm samples and the genome coordinates will be used as parameters for the VCF export. The variant data is stored in an ORACLE RDBMS (version 12c) using columnar partitioning storage and compressed in-memory-population to provide fast access to the marker data from the SNP browser frontend. The backend was implemented with Grails (Smith and Ledbrook, 2009) in version 3.3.8 and the frontend was implemented with ReactJS^1^ in version 16.2.0. The communication between both was realized with a RESTful API.

#### Interactive Scatterplots for Results of Dimensionality Reduction

Exploring population structure and its correlates by dimension reduction methods such as PCA is a commonly used method in population genetics. BRIDGE includes an interactive visualization of the genetic diversity space defined by PCA and t-SNE, allowing selection of dimensions for display, zooming, lasso selection and highlighting accessions according to their country of origin or phenotypic attributes (Figure 3C). The precalculated results of dimensionality reduction methods are stored in the Oracle RDBMS. A lasso selection of germplasm samples inside the scatterplots automatically highlights the countries of origin on the world map and aggregates counts and percentage shares for each country in a tabular summary. Conversely, it is possible to highlight the respective germplasm samples in the diversity space based on a selection of a single country or multiple countries. This enables to inspect the population structure and geographic distribution in an explorative and interactive manner. Color schemes of the scatterplots can be changed by the user according to three options. The first color scheme option uses the user-defined tagging colors of the particular saved germplasm collections. Germplasm, which is not included in any collection, is colorized black. The second color scheme option colorizes the germplasm samples according to their Hamming distance (Hamming, 1950) to the Morex reference genome based on the SNP data. The third option colorizes the dots according to the values of phenotypic traits. The user interface provides a control handle to change the currently colorized trait. The color scheme can be changed by the user to meet personal preferences. This colorization mode handles ordinal and interval scaled trait variables. In addition, the values of the phenotypic traits are displayed on the world map summarized by individual countries. The scatterplot matrix visualization allows the inspection of the first four principal components of PCA results while providing the ability for lasso selections and the highlighting of samples according to their collection tagging color (Figure 3F).

#### Interactive World Map

The interactive political world map provides a geographical visualization for those genotypes that have geographical coordinates of their collection site available in their passport data (Figure 3D). Furthermore, it allows a lasso selection of genotypes, which can be saved as a named germplasm collection. Vice versa, germplasm samples included in a saved germplasm collection are automatically highlighted with their respective tagging color.

#### Interactive Manhattan Plots of Genome Wide Association Studies (GWAS)

The correlation between phenotypic traits and genotypic information can be explored in the zoomable Manhattan plot visualization (Figure 3E). By clicking on a data point of a variant *p*-value the application automatically jumps to that variant position in the embedded SNP browser. The trait to be displayed can be switched by a select box.

#### VCF Export

This module allows the parameterized export of a subset of the underlying SNP matrices (Figure 4I). The export parameter form consists of input fields for the SNP data type, chromosome, start position, end position and accession numbers. It can be completed manually or filled with the corresponding parameters of the current viewport of the SNP browser. For one or more genotypes, markers within a single chromosome can be exported as a VCF file in version 4.0 (Danecek et al., 2011). The VCF export is limited to 1,000,000 data points due to performance reasons. This allows for example to export 1,000 SNP’s for 1,000 genotypes or 100,000 SNP’s for 10 genotypes.

#### MIAPPE-Compliant ISA-Tab Export

Beside the export of genetic information via VCF files, it is also possible to export the phenotypic data records in the ISA-Tab format (Sansone et al., 2012), which combines a machine-readable and a textual human-readable representation (Figure 4J). The observation scores used for phenotyping are described in an embedded “Trait Definition File.” Thus, the provided metadata complies with the version 1.1 of the MIAPPE standard (Krajewski et al., 2015; Ćwiek-Kupczyńska et al., 2016; Papoutsoglou et al., 2020). Due to the strong diversity of phenotypic research data, the standard was initially developed to explain the minimal information that is necessary to describe plant phenotypic experiments. MIAPPE is still under active development^2^. For a more convenient use, BRIDGE offers the possibility to choose between a full data export, which includes the corresponding digital spike image files physically and a metadata-only download with persistent URLs to access the images online.

#### Germplasm Order Service

Saved germplasm collections can directly be transferred to the IPK genebank information system (GBIS) (Oppermann et al., 2015) to get further information or order seeds of accessions of interest (Figure 4K). The transfer is implemented via an HTTP call to the GBIS RESTful API, which checks whether a sufficient amount of seeds is stored at IPK genebank and is available for distribution under the terms of the IPK Genebank Material Transfer Agreement (SMTA). Accessions with insufficient numbers of seeds on stock are automatically excluded but displayed as a list to the user including the reasons for exclusion (e.g., limited seed stock). Users can then fill out the order form in GBIS to receive the desired seed material. We would like to note, that single-seed descent material used throughout the BRIDGE experiments cannot be ordered at present. Material will then be procured from the regular stocks of the IPK genebank. Seeds from accessions that are not maintained at IPK genebank [e.g., accessions from the Swiss and Chinese genebank included in Milner et al. (2019) study] cannot be ordered through GBIS but need to be placed to the respective genebank.

#### Interactive Help

One important task when designing a graphical user interface (GUI) is the conception of menu elements, search fields, buttons and the general layout. Complex applications like BRIDGE that are comprised of many different modules can be overwhelming for first-time users. We therefore minimized the number of interactive elements in the GUI exposed to the user at any single point in time. Only the main navigation is always visible on the menu to the left and does not contain nested submenus (Figure 1). Moreover, we integrated an interactive tutorial that guides the process of discovering the potential and power of the application. This discovery is supported by a context-dependent, visual highlighting of control elements and a textual description of their function. It is implemented with the help of the IntroJS library (Arasteh and Mehrabani, 2013).

## Results

BRIDGE allows an easy-to-use access to exploratory data analysis of passport and genotypic data of a worldwide panel of barley accessions, phenotypic data, related diversity data and downstream research results (Figure 4). Through the use of techniques that users experienced through popular web pages that use world maps or lasso selection like in image editing programs, the barrier to use BRIDGE is considerably lower than in similar systems. Furthermore, much of BRIDGE has been designed with a novice user in mind and we guide them with our interactive help function. Due to the high integration level of the visualization and analysis tools as a single-page application, data sets representing research results can be conveniently and quickly reviewed and analyzed. In comparison to a traditional multi-page application there are no time-consuming page reloads from the web server initiated by actions via navigation links or functional buttons. This leads to an increased user experience when using the application. Hence, the single-page application behaves and feels more like a traditional standalone desktop application with minimal time delay between a user action and the corresponding application response. Another advantage is the prevention of time-consuming and error-prone manual data conversions and data transfers between multiple standalone programs for each data visualization domain.

The identification of plant genotypes that meet certain criteria for adaptation to climatic and agronomic conditions as well as criteria for nutritional traits is a basic requirement for successful cultivation and improvements in breeding. This challenge is multidimensional, as multiple criteria must be met by a single crop genotype. Digital information systems such as BRIDGE can support this identification of suitable crop genotypes by providing convenient access and searchability in this multidimensional data space. Plant scientists might be interested in finding candidates of barley accession with specific genetic variants for a research topic they are working on, while barley breeders are looking for high diversity of target quality traits to complement their breeding panel with promising plant genetic resources. These different user groups have distinct demands for their workflows and subsequently we show how BRIDGE can tailor these demands by presenting selected exemplary use cases. Nevertheless, this is by far not a complete set what can be done theoretically by using the application.

### Exemplary Use Cases

#### Finding Accessions With Characteristic Genotypic and Phenotypic Features

BRIDGE allows the search of germplasm with specific genotypic and/or phenotypic traits. By using the variant filter feature in the “Search germplasm” panel, the user can find germplasm with specific variants for one or more SNPs. The filter setting on variants can be combined with filters for passport data and phenotypic attributes of interest (Figure 1, available under the feature “Search Germplasm” and one starting point to create a user specific sample collection, see Figure 4E). This feature might be useful for breeders as well as for scientists that want to identify genotypes matching certain criteria for crop improvement or similar research questions. Another functionality is the ability to find the corresponding genomic region for a given gene of interest as a function of the integrated SNP browser. A scientist or breeder can then export marker data as VCF files for his germplasm samples of interest to feed this variation data into subsequent analysis steps (Figure 4I).

#### Ordering Germplasm With Specific Attributes of Interest

Through the integration with the IPK Genebank Information System (GBIS), it is possible to order germplasm of interest by transferring individually created germplasm collections to GBIS (Figures 4E,D,K). This is convenient if a user wishes to order, for example, germplasm from one of the predefined core sets or a user-defined collection of germplasm samples identified by prior exploratory data analysis. The integration avoids the manual transfer of germplasm sample lists through export and import, thus reducing the probability for errors.

#### Usage as a Decision Support System for Genebank Data Curation

Mascher et al. (2019) gave one example how BRIDGE was used as a decision support system to improve the data quality of the barley collection of the IPK genebank. The combined and interactive access to passport data and results of genetic clustering algorithms revealed that thousands of Ethiopian accessions in the IPK genebank had a false biological status of “wild” instead of the correct one: “domesticated.” This can be verified in BRIDGE by searching for germplasm with “Ethiopia” as country of origin and with “wild” as biological status (defining filters using the “Search Germplasm” feature, see Figures 1, 4A). The search result must be saved as a user-defined collection (Figures 4D,E). When switching to the PCA plot, it is clearly visible that these wild Ethiopian accessions are located in a cluster of domesticated barley (for filter definition using the feature “Search Germplasm” and for visualization the features “PCA Matrixplot” and “Geographic Origins,” see in Figure 1 and similar visualized like in Figures 4G,H).

#### Data Stewardship as Necessary Precondition for Exploratory Data Analysis

**FIGURE 5:**
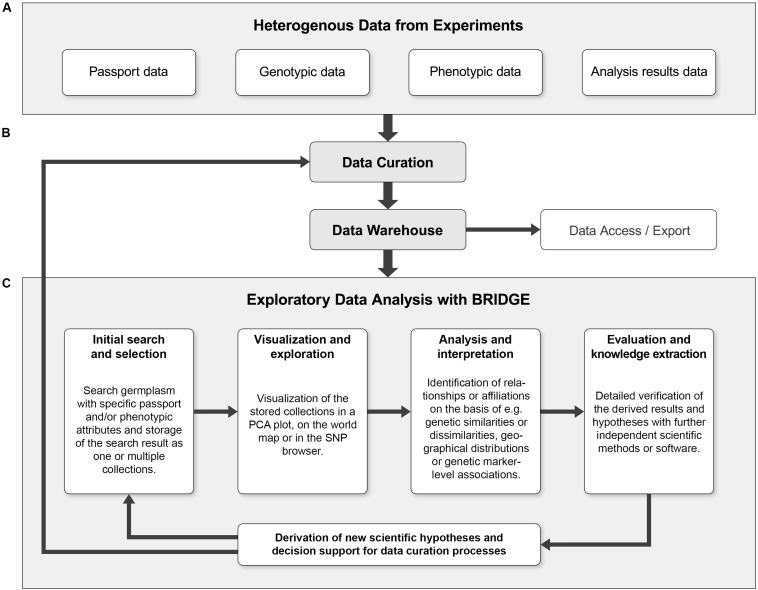
The combination of exploratory data analysis and continuous data curation can form a cycle that leads to continuous quality improvements. **(A)** The heterogeneous primary data from genotyping and phenotyping experiments combined with data of further analysis steps like PCA or GWAS (“Analysis results data”) are collected and serve as initial data sources. **(B)** The input data is subjected to a first curation step and then fed into the data warehouse. The data warehouse allows programmatic access to the data and export of the data in standardized formats. **(C)** The iterative process of exploratory data analysis (EDA) makes it possible to derive new scientific hypotheses without prior assumptions. Furthermore, the result of an EDA iteration can reveal data inconsistencies and thus be a starting point for a subsequent data curation step. Ideally, these processes form a continuous cycle, which can lead to continuous quality improvement of primary research data and derived data. Using an entry point such as the germplasm search, a “named collection” of germplasm to be visualized or explored is created. Users can then search for outliers, clusters or other visual keys in a genetic diversity plot (such as PCA). When a sample or a set of samples of specific interest is identified and highlighted by a lasso selection, it is possible to save this subset as a new collection and look for further details, e.g., via the SNP profiles in the SNP browser. This detailed look into the data could be enough to find something unusual that might be worth further experimentation or lead to new scientific findings.

An integrated exploratory analysis of phenotypic and genotypic data is possible if used plant material and probes have been consequently processed in a FAIR-compliant laboratory process. In BRIDGE, we applied homogenized protocols for phenotyping, genotyping, data analysis and storage tracking, accompanied by a strict data stewardship. This procedure enables the return of investment of the efforts for data quality management, which is essential for the integrated and explorative analysis of primarily heterogeneous data. In addition, the iterative process of exploratory data analysis enables the continuous support of data curation within the framework of data stewardship principles (Wilkinson et al., 2016; Figure 5).

### Comparison With Similar Web Portals

Integrative applications for web-based exploration of data in the data domain of genotyping and phenotyping data are scarce especially those focussing on plant genetic resources. In the following, we compare BRIDGE to two well-established barley specific web portals (Germinate3 and T3/Barley) and present the individual strengths and weaknesses. Results are summarized in Table 4. We note that there are more systems available, especially when considering other organisms, and this list should not be regarded as complete.

**TABLE 4:** Comparison between BRIDGE, Germinate3, and T3/Barley.

Resource name	Resource type	Custom collections	Interactive brushing and linking	Integrated visualizations	Interactive plots	Data export
Germinate3	Data warehouse	(✓)	×	✓	(✓)	CSV, Flapjack
T3/Barley	Data warehouse	×	×	✓	×	CSV, Flapjack
BRIDGE	Visual analytics webtool	✓	✓	✓	✓	ISA-tab, VCF, CSV

*The features of the resources are compared based on: resource type, the ability to subset the data into custom collections, if the concept of “interactive brushing and linking” has been implemented, the ability to generate graphical results, the interactivity of generated plots, and the data export formats. The “✓” shows a full compliance, while “(✓)” shows a partial compliance and the “×” shows no compliance.*

#### Germinate3

One application framework for which a barley instance is available is Germinate3 (Shaw et al., 2017). Compared to BRIDGE, it shares some of the features and behaves more like a traditional table-based data warehouse than a visual analytics driven application. Thus, the individual tables do not interact with each other and can be regarded as static instances. It also follows the principle of an integrated and uniform user interface and the concept of sample collections, called “groups.” A functionality shared with BRIDGE is the geographical world map with the possibility to pick germplasm samples into a collection by drawing a line around them. A combined search for passport and phenotypic data as well as a seamlessly integrated possibility to visualize marker data is not implemented. However, marker data can be exported to different file formats with one being the proprietary Flapjack file format (Milne et al., 2010). This workflow decelerates the speed for cycles of exploratory data analysis because the data has to be downloaded, then imported into the standalone Flapjack software and finally has to be analyzed until new knowledge can be generated. This in turn decreases the user experience and is prone to produce errors from either shifting platforms or from version conflicts. Germinate3 also provides the ability to render scatterplots which is available for phenotypic data. Furthermore, there is no possibility to export genotypic and phenotypic data in standardized file formats. A possibility to visualize the results of GWAS by Manhattan diagram is not implemented. Nevertheless, Germinate3 can be described as a generic database solution for plant genetic resources. Several different plant species have been set up including barley, chickpea, eggplant, maize and wheat (with others in active development). While BRIDGE offers interactive visual analytics tools Germinate3 is more suited for users that prefer table-based analysis.

#### T3/Barley

T3/Barley^3^ is the successor of The Hordeum Toolbox (Blake et al., 2012). It has a multitude of functions and a complex user interface with a main navigation consisting of several submenus. Even though the portal offers a lot of functionality, the user is overwhelmed by many acronyms and abbreviations that are not explained. For the visualization of genetic diversity data, the portal offers an export function to create files for the standalone genome viewer Flapjack (Milne et al., 2010). Data export is possible via CSV formats which makes downstream analysis challenging as it is the user’s responsibility to transform the data into appropriate standard formats. In contrast to BRIDGE, users of the T3/Barley resource need a deep understanding of the system. New users of this resource may have difficulty obtaining meaningful results because many tools and visualizations are hidden behind layers of parameter selection screens.

## Discussion

In the following, we will discuss the key benefits of information systems and tools such as BRIDGE in the context of the growing volume of heterogeneous genomic and phenomic data, the integration of new data sets and possible useful functional enhancements.

### Integration and Information Retrieval of Growing Genomic and Phenomic Data Sets

Technology breakthroughs of the last decade like next generation sequencing and high-throughput phenotyping have significantly changed the scientific landscape (Elshire et al., 2011; Pieruschka and Schurr, 2019). The speed and amount of data generation was accelerated to a new level and it is becoming more and more challenging to analyze and visualize scientific findings in a comprehensive way. Many software tools have been developed in recent years with similar goals: Frustration-free access to constantly growing life science data sets for exploration, analysis and interoperability. As systems for the integration and linkage of heterogeneous data sets, they can greatly help to avoid the problem of not seeing the forest for the trees and offer a low-barrier assistance to extract hidden treasures from the “sea of bioinformatics data” (Roos, 2001), a challenge that has existed for at least 20 years. Furthermore, such web portals can serve as a unified entry point for validation, recapitulation and export of PGR data sets as well as an “exploratory data analysis playground” for generating new research ideas and hypotheses (de Mast and Kemper, 2009).

In addition, application concepts such as BRIDGE can be regarded as proof-of-concepts and blueprints for the software-supported transformation of genebanks into bio-digital resource centers, thus helping to close the gap between the preservation of crop diversity in genebanks and plant breeding (Mascher et al., 2019). This support can be achieved by leveraging the power of visual analytics and exploratory data analysis to keep track of the ever-growing volume of PGR related data sets. We highlighted one use case, where such exploratory data analysis could be applied to directly increase the data quality by correcting falsely labeled wild germplasm. Thus, processes need to be defined which can directly annotate or change the passport information in information systems managing PGR data. In the future, it will be possible in BRIDGE to switch between either historical passport data or passport data updated by the continuously ongoing data curation process.

### Integration of New Data Sets

As more and more large collections of genetic material are being molecularly characterized using NGS technology (Weigel and Mott, 2009; The 3, 000 rice genomes project, 2014; Mascher et al., 2019), one possible step is to integrate genomic data of germplasm from other genebanks. This would increase the overall allelic diversity of the entire barley panel and thus increase the usefulness in finding genotypes with specific or rare variants. Many challenges, have yet to be addressed to fully integrate different data sets. Currently, variants are called in regards to a single reference genome and will only record polymorphisms detected in the analyzed population. An integration of several such data sets could lead to a contrasting result for individual variants, with a certain variant only recorded in one population while another variant is reported in another. In addition, the version of reference genome sequence would have to be identical in order to compare or integrate different data sets.

Since a single reference genome sequence cannot explain all possible variants in a given species, researchers have started to study variants on a larger scale with regard to the so-called pan-genome. It was previously reported that much variation in elite breeding material has been lost due to the domestication bottleneck (Tanksley and McCouch, 1997; Hyten et al., 2006; Kilian et al., 2006; Zhu et al., 2007). Especially in resistance breeding, the use of PGR or plant wild relatives in breeding programs is a promising field for the introduction of new resistance genes. Additional reference-quality genome sequences will allow researchers and plant breeders to better exploit molecular marker data in diverse germplasm. We imagine the possibility to easily switch the reference genome sequence in the SNP browser of BRIDGE or even the integration of a pan-genome browser will help with such an analysis.

Another aspect could be the inclusion of sequences from other sequencing techniques such as exome capture resequencing (Mascher et al., 2013) or WGS data from genotypes already used in BRIDGE to increase sequencing depth. Such an increase would be beneficial for multiple objectives, such as reducing the proportion of missing data and the ability to call more variants. In the case of WGS data this would also allow comparative analysis of structural variations (Zhou et al., 2018). At the time of writing, it is planned to sequence between 100 and 1000 genotypes belonging to one of the core sets for BRIDGE (Monat et al., 2019). These accessions will be subjected to WGS and will help to further characterize the genomic diversity of barley germplasm.

### Useful Feature Enhancements

We plan to improve the current implementation of the SNP browser. One feature we believe requires such an improvement is the feature track. Currently, only the gene boundaries are drawn and a mouse overlay displays the gene name. It would be useful to see the intron-exon structure as well as different isoforms of genes to determine if a variant could have an effect on the coding sequence. In addition, changes to the protein sequence should be visible at a glance. The feature track could also be used to highlight QTL regions where users can examine the annotated genes and search for causal candidates for an observed phenotype. Such a QTL region would be defined by a collection of SNP markers. A lasso selection in a GWAS Manhattan plot or a range selection in the SNP browser could be a starting point to generate such a collection. We are also considering improving the functionality of the statistics track. At the moment, both the SNP coverage and the minor allele frequency are precalculated for the entire data set. However, it would be useful to dynamically calculate these metrics for a selected germplasm subset. Furthermore, the differences in metrics between the entire data set and a collection might be helpful, and we are experimenting with different methods to visualize that. It would also be very useful to be able to group similar SNP profiles of different genotypes together. This could provide a haplotype based view on the selected collection and a way to reduce visual noise. A very useful feature for the SNP browser would be the ability to dynamically filter variants according to various criteria like e.g., minor allele frequency, number of heterozygous calls, quality of the SNP and number of supporting reads.

We also want to simplify the handling of germplasm collections throughout BRIDGE. Currently, only one list of germplasm collections can be managed at a time. A potential worksheet feature would be helpful to be able to manage several different lists at the same time. This feature would allow the organization of multiple and different sets of sample collections to address different research topics or explorative workflows. Concerning the germplasm search, we hope to extend this function to include genotyping data that allows a combined search not only for passport and phenotypic data, but also for genotypic data.

Another interesting and promising feature would be an online calculation for dimensionality reductions, such as PCA or t-SNE on the SNP data. Based on an individually generated subset of germplasm samples, this approach would allow for a semi-automated exploration of the population structure at a fine-grained resolution, e.g., by applying a PCA to a local cluster of genotypes. Due to the large size of the SNP data and the resulting computational load, these calculations cannot be performed client-side on the user’s device. Instead these calculations would have to be performed on the web server, which in turn would have to be sufficiently powerful in terms of CPU and RAM. Alternatively, a calculation in an elastic cloud environment could be considered that can react flexibly to the required calculation effort. Due to the longer calculation times in general and the potentially parallel occurrence of a calculation request through simultaneous actions by multiple users, a job queue would be required. This queue could process the individual calculations in turn and send the user a notification that the calculation is complete.

## Conclusion

BRIDGE is an application concept and implementation for the visual analytics driven exploration of data of plant genetic resources (PGR), mainly stored at the German Federal *ex situ* genebank at IPK Gatersleben. We presented the benefits of a quality curated data warehouse of integrated genomics and phenomics data based on a deeply genotyped and phenotyped worldwide barley germplasm collection of 22,626 genotypes. The genotypic and phenotypic data is extended with linked downstream analysis data like GWAS results and dimensionality reduction results of the SNP data. In particular, we demonstrated the benefit of multiple entry points for germplasm search, analysis, knowledge extraction and data export allowing plant scientists and plant breeders to extract domain specific information of personal interest. Users can benefit from this curated combination of legacy and newly derived data by the ability to order barley accessions of specific relevance and by incorporating these ordered PGRs in their own research or breeding efforts. Furthermore, the user can benefit by exporting subsets of the provided data in common file formats like VCF for data of genetic variants or MIAPPE-compliant ISA-Tab archives for phenotypic data. Application concepts like BRIDGE can act as a proof-of-concept for the software-aided transformation of genebanks into bio-digital resource centers allowing to close the gap between the conserved crop diversity, plant breeding and research. Moreover, they can serve as a first entry point for data curation and scientific hypothesis generation. Feedback from IPK and close collaboration partners has revealed the potential a number of meaningful and useful possibilities and ideas for extending the functionality of the system, which will be implemented in future versions of BRIDGE.

## Data Availability Statement

Publicly available datasets were analyzed in this study. This data can be found here: https://www.nature.com/articles/s41588-018-0266-x#data-availability, https://doi.org/10.5447/IPK/2018/9, and https://doi.org/10.5447/IPK/2018/10.

## Author Contributions

NS, MM, and US designed the study. NS supervised the experiments. MM analyzed the data. PK designed and developed the main parts of the application software. MB, MM, and ML designed and MB implemented the SNP browser. DS, PK, SB, ML, and DA curated, analyzed, and imported data. ML developed the data storage concept. DA implemented the ISA-Tab export API. US and ML supervised the development of the portal. PK, SB, DA, US, and ML wrote the manuscript with contributions from all co-authors. All authors read and approved the final manuscript.

## Conflict of Interest

The authors declare that the research was conducted in the absence of any commercial or financial relationships that could be construed as a potential conflict of interest.

## Footnotes

1 https://reactjs.org

2 https://www.miappe.org

3 https://triticeaetoolbox.org/barley
